# Association of Urinary Nitrate With Diabetes Complication and Disease-Specific Mortality Among Adults With Hyperglycemia

**DOI:** 10.1210/clinem/dgac741

**Published:** 2022-12-28

**Authors:** Wenbo Jiang, Jia Zhang, Ruiming Yang, Xinyi Sun, Huanyu Wu, Jiacheng Zhang, Siyao Liu, Changhao Sun, Lifang Ma, Tianshu Han, Wei Wei

**Affiliations:** Department of Nutrition and Food Hygiene, The National Key Discipline, School of Public Health, Harbin Medical University, Harbin 150081, P. R. China; Department of Cardiology, The First Affiliated Hospital of Harbin Medical University, Harbin 150081, China; Department of Nutrition and Food Hygiene, The National Key Discipline, School of Public Health, Harbin Medical University, Harbin 150081, P. R. China; Department of Nutrition and Food Hygiene, The National Key Discipline, School of Public Health, Harbin Medical University, Harbin 150081, P. R. China; Department of Nutrition and Food Hygiene, The National Key Discipline, School of Public Health, Harbin Medical University, Harbin 150081, P. R. China; Department of Nutrition and Food Hygiene, The National Key Discipline, School of Public Health, Harbin Medical University, Harbin 150081, P. R. China; Department of Nutrition and Food Hygiene, The National Key Discipline, School of Public Health, Harbin Medical University, Harbin 150081, P. R. China; Division of Epidemiology, Biostatistics and Environmental Health, School of Public Health, University of Memphis, Memphis, TN 38152, USA; Department of Nutrition and Food Hygiene, The National Key Discipline, School of Public Health, Harbin Medical University, Harbin 150081, P. R. China; Department of Pharmacology, College of Pharmacy Key Laboratory of Cardiovascular Research, Ministry of Education, Harbin Medical University, Harbin 150081, P. R. China; Department of Nutrition and Food Hygiene, The National Key Discipline, School of Public Health, Harbin Medical University, Harbin 150081, P. R. China; Department of Nutrition and Food Hygiene, The National Key Discipline, School of Public Health, Harbin Medical University, Harbin 150081, P. R. China; Department of Geriatrics, The First Affiliated Hospital of Harbin Medical University, Harbin 150001, P. R. China

**Keywords:** urinary nitrate, diabetes complications, all-cause, disease-specific mortalities, hyperglycemia

## Abstract

**Context:**

The hyperglycemia condition disrupts metabolism of nitrate/nitrite and nitric oxide, and dietary nitrate intake can restore nitric oxide homeostasis.

**Objective:**

This study aims to examine whether urinary nitrate is associated with diabetes complications and long-term survival among people with hyperglycemia.

**Methods:**

A total of 6208 people with hyperglycemia who participated in the National Health and Nutrition Examination Survey from 2005 to 2014 were enrolled. Diabetes complications included congestive heart failure, coronary heart disease, angina, stroke, myocardial infarction, diabetic retinopathy, and nephropathy. Mortality was obtained from the National Death Index until 2015. Urinary nitrate was measured by ion chromatography coupled with electrospray tandem mass spectrometry, which was log-transformed and categorized into tertiles. Logistic regression models and Cox proportional hazards models were respectively performed to assess the association of urinary nitrate with the risk of diabetes complications and disease-specific mortalities.

**Results:**

After adjustment for potential confounders, including urinary perchlorate and thiocyanate, compared with the participants in the lowest tertile of nitrate, the participants in the highest tertile had lower risks of congestive heart failure (odds ratio [OR] 0.41; 95% CI, 0.27-0.60) and diabetic nephropathy (OR 0.50; 95% CI, 0.41-0.62). Meanwhile, during a total follow-up period of 41 463 person-years, the participants in the highest tertile had lower mortality risk of all-cause (hazard ratio [HR] 0.78; 95% CI, 0.62-0.97), cardiovascular disease (CVD) (HR 0.56; 95% CI, 0.37-0.84), and diabetes (HR 0.47; 95% CI, 0.24-0.90), which showed dose-dependent linear relationships (*P* for nonlinearity > 0.05). Moreover, no association between nitrate and cancer mortality was observed (HR 1.13; 95% CI, 0.71-1.80).

**Conclusion:**

Higher urinary nitrate is associated with lower risk of congestive heart failure and diabetic nephropathy, and lower risk of all-cause, CVD, and diabetes mortalities. These findings indicate that inorganic nitrate supplementation can be considered as a supplementary treatment for people with hyperglycemia.

Nitrate is a natural chemical substance that exists widely in the environment and food. Accumulating evidence has shown that its major source in the general population is from drinking water and food, including plants, vegetables, fruits, dairy products, and meats (1, 2). Measurement of urinary nitrate has been frequently used to assess its intake levels in humans (3). Some in vivo studies in humans and mammals indicate that circulating nitrite rather than nitrate reflects endothelial-dependent NO synthesis in humans and mammals. It is clearly established that the major urinary metabolite of nitric oxide (NO) is nitrate (4). Urinary and serum nitrate levels were associated with the progression of infection, gastroenterological conditions, hypertension, renal and cardiac disease, inflammatory diseases, transplant rejection, diseases of the central nervous system, and others (5). It has been documented that circulating perchlorate and thiocyanate have internal competitive effects on nitrate metabolism (6). Accumulating evidence has suggested that perchlorate, nitrate, and thiocyanate could competitively inhibit thyroid iodine uptake and exert potential adverse effects on thyroid function. Therefore, when analyzing one of the health effects, it is necessary to consider the influence of the other 2 factors.

Currently, there are reports from many animal and human studies that have examined the health impacts of perchlorate, nitrate, and thiocyanate, and the controversy is largely from the health effects of nitrate among people with hyperglycemia. The prevalence of diabetes has increased dramatically over the past 3 decades (7). It has been demonstrated that the hyperglycemia condition can disrupt the metabolism of nitrate/nitrite and NO through increased oxidative stress, accumulation of advanced glycation end products, increased asymmetric dimethylarginine, and decreased L-arginine bioavailability and NO synthase, contributing to the consequent complications of hyperglycemia in metabolism and vascular (8, 9). Moreover, a previous study has demonstrated that with progressive development of hyperglycemia, more and more superoxides accumulate, which further inhibits endothelial nitric oxide synthase, forming a vicious cycle (10). Therefore, inorganic nitrate supplementation is considered to be a supplementary treatment for patients with diabetes because animal studies found that dietary nitrate intake can restore NO homeostasis and maintain steady-state NO levels under the context of disrupted metabolism of NO (11). However, while some randomized controlled trials of short-term interventions (2 weeks to 6 months) with supplementary nitrate have shown that dietary nitrate supplementation among patients with prediabetes or diabetes can significantly affect the level of serum glucose or insulin in the body, others have shown that nitrate supplementation has no significant effect on blood pressure, insulin sensitivity, and other metabolic parameters in patients. These results were inconsistent, probably because these studies mainly focused on the hypoglycemic effects with relative short intervention periods or small sample sizes, which makes it difficult to clarify the potential health impacts on the development of diabetes complication and long-term survival (12-17). More importantly, evidence from large or long-term observation studies for clarifying the association of nitrate with diabetes complications and long-term survival among people with hyperglycemia is still lacking.

To fill this gap, this study aimed to simultaneously examine the association of urinary perchlorate, nitrate, and thiocyanate with complications of diabetes and mortality of all-cause, cardiovascular disease (CVD), and diabetes to elucidate whether and how nitrate is associated with the complications of diabetes and long-term survival among patients with hyperglycemia.

## Methods

### Study Population

The National Health and Nutrition Examination Survey (NHANES) is a multistage stratified sampling study, which aims to assess the health and nutrition status among the noninstitutionalized civilian population of the United States (18). This study enrolled the hyperglycemic adults ≥ 18 years old who participated in NHANES from 2005 to 2014. The standard of the American Diabetes Association for hyperglycemia was used, as a self-reported diabetes diagnosis history, and/or fasting glucose level ≥ 5.6 mmol/L, and/or HbA1c level >5.7% (19). After excluding the participants who had missing information of urinary perchlorate, nitrate, thiocyanate, and creatinine, 6208 participants with hyperglycemia, including 3348 men and 2860 women were included in this study. The institutional review board approval of the National Center for Health Statistics and written informed consent was obtained before data collection.

### Dietary Assessment

Food intake information over 2 discontinuous days was collected through 2 nonconsecutive 24-hour dietary recall interviews. The first one was conducted in person and the other was performed over the phone 3 to 10 days later. Mixed foods were subdivided into their constituent parts using the Department of Agriculture's Food and Nutrition Database and My Pyramid Equivalent Database 2.0, which were further categorized into 18 food groups. The dietary information in NHANES has been widely used in previous nutritional studies.

### Main Exposures

Urine specimens were collected and stored under appropriate conditions until they were shipped to National Center for Environmental Health in the Centers for Disease Control and Preventions for further testing. Urine samples were stored in freezers at temperature ≤ −70 °C until measurement. Urinary perchlorate, nitrate, and thiocyanate were quantified by ion chromatography coupled with electrospray tandem mass spectrometry with the limits of detection (LODs) being 0.05 ng/mL, 700 ng/mL, and 20 ng/mL, respectively. Measurements below the LODs were replaced with the values of LOD divided by the square root of 2. An automated colorimetric method on a Beckman Synchron AS/ASTRA clinical analyzer (Beckman Instruments Inc., Brea, CA) was used to measure urinary creatinine levels. All the assays met the Laboratory Science's quality control and quality assurance performance criteria for accuracy and precision in the National Center for Environmental Health (20).

### Main Outcomes

The main outcome variables were diabetes complications and disease-specific and all-cause mortality. The diabetes complications included congestive heart failure, coronary heart disease, angina, stroke, myocardial infarction, diabetic retinopathy, and diabetic nephropathy. The participants were considered to have congestive heart failure, coronary heart disease, angina, stroke, myocardial infarction, or diabetic retinopathy if they separately answered yes to the questions “Has a doctor or other health professional ever told you that you had congestive heart failure/coronary heart disease/angina/stroke/myocardial infarction (heart attack)/diabetic retinopathy?” These questions were included in the medical conditions part of the NHANES, and the participants’ diseases condition were based on the medical history record by their clinicians or medical departments. The estimated glomerular filtration rate (eGFR) was calculated based on the equation developed by the Chronic Kidney Disease Epidemiology Collaboration (21). The diabetic nephropathy was defined as a clinical syndrome in the patients characterized by persistent albuminuria (>300 mg/day or >20 μg/min) or estimated glomerular filtration rate < 60 mL/min/1.73 m^2^ at 2 out of 3 examinations within 3 to 6 months (22). Prevalent cases of diabetes complications including 315 cases for congestive heart failure, 373 cases for coronary heart disease, 253 cases for angina, 341 cases for stroke, 409 cases for myocardial infarction, 347 cases for diabetic retinopathy, and 1616 cases for diabetic nephropathy were documented.

The all-cause, CVD, diabetes, and cancer deaths were determined by the National Death Index, a highly reliable resource widely used for death identification. The ICD-10 is used to determine disease-specific death, among which ICD-10 codes I00–I09, I11, I13, I20–I51, or I60–I69 were defined as CVD mortality, codes E10–E14 were defined as diabetes mortality, and codes I19-I43 were defined as cancer mortality. During a total follow-up period of 41 463 person-years, incident cases of disease-specific mortalities were documented, including 838 deaths, comprising 268 deaths due to CVD, 131 deaths due to diabetes, and 191 deaths due to cancer.

### Confounder Assessments

The confounders included age (years), gender (men or women), race (non-Hispanic White/non-Hispanic Black/Mexican American/other), education level (<9th grade, 9th-11th grade, high school graduate, GED or equivalent, some college or Associate in Arts degree, or college graduate or above), annual family income (<$20 000, $20 000 to $45 000, $45 000 to $75 000, $75 000 to $100 000, or >$100 000), regular exercise habits (yes/no), smoking (yes/no), drinking (yes/no), body mass index (BMI, kg/m^2^), daily energy intake (kcal/d), Alternative Healthy Eating Index (AHEI), systolic blood pressure (SBP) (mmHg), diastolic blood pressure (DBP) (mmHg), fasting plasma glucose (mmol/L), glycated hemoglobin (HbA1c) (%), the homeostatic model assessment of insulin resistance (HOMA-IR), plasma triglycerides (TG, mmol/L), plasma total cholesterol (TC, mmol/L), high-density cholesterol lipoprotein (HDL-C, mmol/L), low-density cholesterol lipoprotein (LDL-C, mmol/L), urinary creatinine, and drug use for controlling glucose/hypertension/dyslipidemia (yes/no).

### Statistical Analysis

The baseline characteristics in terms of demographics, lifestyle, dietary behavior, and biochemical indices were presented as mean (SD) and numbers (percentage). The urinary levels of perchlorate, nitrate, and thiocyanate were log-transformed and categorized into tertiles. General linear models adjusting for age and gender, and the chi-squared test were used to compare the differences for baseline characteristics by tertiles of urinary perchlorate, nitrate, and thiocyanate, respectively. Logistic regression models were performed to evaluate the odds ratios (ORs) and 95% CI for the association of urinary perchlorate, nitrate, and thiocyanate with the risk of diabetes complications. Cox proportional hazards models were performed to evaluate the association of urinary perchlorate, nitrate, and thiocyanate with mortality from all causes, CVD, diabetes, and cancer. Survival time was months between NHANES interview date and death or census date (December 31, 2015). Restricted cubic spline was used to visualize the dose-response association of the significant association found in the logistic regression or Cox proportional hazards models by setting 4 knots at 5th, 25th, 75th, and 95th percentiles, and analysis of variance (ANOVA) was used to examine the linear or nonlinear relationship of the spline. A series of confounders were also controlled in regression models and restricted cubic spline, which included age, gender, race, education, income, smoking, drinking, regular exercise habits, BMI, daily energy intake, overall dietary quality, drug use for controlling hyperglycemia, hypertension and dyslipidemia, SBP, DBP, urinary creatinine, fasting plasma glucose, HbA1c, HOMA-IR, TG, HDL-C, LDL-C, and TC.

All statistical analyses were conducted by R 4.0.2, and a statistically significant level was defined as a *P* value < 0.05.

### Sensitivity Analysis

Four sets of sensitivity analyses were performed to evaluate the robustness of the association between urinary nitrate and diabetes complications, disease-specific and all-cause mortality. In set 1, participants who had a follow-up period of less than 2 years were excluded to assess the impact of acute illness or accident on results. In set 2, we excluded the participants who had extreme values (>99% and/or <1%) of urinary creatinine, perchlorate, nitrate, and thiocyanate. In set 3, corrected urinary perchlorate, nitrate, and thiocyanate values were calculated by dividing by urinary creatinine, which was further used in the statistical model to exclude the impact of urine dilution on the results. In set 4, we evaluated whether age, gender, BMI, smoking, and drinking had a modification effect on these relationships.

## Results

### Baseline Characteristics

Table 1 shows the differences for the baseline characteristics in terms of demographic, anthropometric, lifestyle, dietary behavior, and biochemical indicators across tertiles of baseline urinary perchlorate, nitrate, and thiocyanate. Participants with higher urinary perchlorate were more likely to be men and non-Hispanic White with lower SBP, HDL-C, and LDL-C and higher HOMA-IR and urinary creatinine (all the *P* < 0.05). Meanwhile, participants with higher urinary nitrate were younger and more likely to be men, and at higher levels of education and income with a higher percentage of smoking rate, drinking rate, and lower levels of SBP, fasting glucose, HOMA-IR, and HDL-C, as well as lower risk of hypertension (all the *P* < 0.05). Similarly, participants with higher urinary thiocyanate were younger and more likely to be men and non-Hispanic White with a higher percentage of smoking rate and drinking rate, and lower levels of SBP, fasting glucose, HDL-C, as well as lower risk of hypertension (all *P* < 0.05).

**Table 1.** Characteristics of study participants according to tertiles of urinary perchlorate, nitrate, and thiocyanate

Characteristics	Urinary perchlorate (ng/mL)				Urinary nitrate (ng/mL)				Urinary thiocyanate (ng/mL)			
	T1	T2	T3	*P* value	T1	T2	T3	*P* value	T1	T2	T3	*P* value
Age, years	56.5 (15.5)	56.9 (15.6)	56.4 (15.6)	0.543	60.6 (15.0)	56.2 (15.4)	53.1 (15.4)	<0.001	60.8 (15.2)	57.1 (15.7)	52.0 (14.5)	<0.001
Men, N (%)	978 (47.5)	1153 (55.5)	1217 (58.8)	<0.001	944 (45.6)	1168 (56.6)	1236 (59.6)	<0.001	968 (46.8)	1095 (52.8)	1285 (62.2)	<0.001
Non-Hispanic White, N (%)	845 (41.0)	887 (42.7)	933 (45.1)	<0.001	919 (44.4)	850 (41.2)	896 (43.2)	<0.001	746 (36.1)	903 (43.4)	1016 (49.2)	<0.001
BMI, kg/m^2^	30.3 (7.0)	31.3 (7.6)	31.0 (6.9)	<0.001	30.2 (6.8)	31.3 (7.7)	31.1 (7.0)	<0.001	29.9 (6.3)	31.4 (7.2)	31.2 (7.9)	<0.001
Current smoking, N (%)	518 (25.1)	487 (23.4)	510 (24.6)	0.424	359 (17.3)	494 (23.9)	662 (31.9)	<0.001	143 (6.9)	192 (9.3)	1180 (57.1)	<0.001
Current drinking, N (%)	1362 (66.1)	1415 (68.1)	1457 (70.4)	0.011	1326 (64.1)	1417 (68.7)	1491 (71.9)	<0.001	1228 (59.4)	1429 (68.9)	1577 (76.4)	<0.001
Regular exercise, N (%)	305 (14.8)	307 (14.8)	301 (14.5)	0.969	295 (14.3)	306 (14.8)	312 (15.0)	0.758	291 (14.1)	315 (15.2)	307 (14.9)	0.583
College graduate or above, N (%)	320 (15.5)	363 (17.5)	358 (17.3)	0.321	304 (14.7)	361 (17.5)	376 (18.1)	0.003	369 (17.8)	402 (19.4)	270 (13.1)	<0.001
Annual household Income>$100 000, N (%)	160 (7.8)	182 (8.8)	196 (9.5)	0.652	128 (6.2)	185 (9.0)	225 (10.8)	<0.001	163 (7.9)	199 (9.6)	176 (8.5)	<0.001
SBP, mmHg	129.6 (20.6)	128.5 (19.0)	127.9 (18.2)	<0.001	131.6 (21.9)	128.0 (18.0)	126.4 (17.3)	<0.001	130.9 (20.5)	128.4 (19.3)	126.8 (17.9)	<0.001
DBP, mmHg	70.1 (14.1)	69.8 (13.9)	69.9 (13.9)	0.831	68.4 (15.1)	70.9 (13.5)	70.7 (13.2)	<0.001	68.7 (14.8)	69.8 (13.9)	71.4 (13.1)	<0.001
FPG, mmol/L	6.65 (2.58)	6.72 (2.43)	6.76 (2.19)	0.525	6.92 (2.93)	6.69 (2.26)	6.51 (1.95)	<0.001	6.89 (2.80)	6.70 (2.34)	6.54 (2.06)	0.002
HbA1c, %	6.26 (1.39)	6.28 (1.34)	6.27 (1.20)	0.911	6.39 (1.45)	6.28 (1.33)	6.14 (1.13)	<0.001	6.39 (1.46)	6.25 (1.23)	6.16 (1.22)	<0.001
HbA1c, mmol/mol	44.9 (15.2)	45.1 (14.7)	45.0 (13.1)	0.911	46.3 (15.9)	45.1 (14.5)	43.6 (12.3)	<0.001	46.4 (15.9)	44.8 (13.5)	43.8 (13.4)	<0.001
HOMA-IR	3.64 (5.30)	3.90 (4.25)	4.32 (6.65)	0.011	3.81 (5.54)	4.27 (6.60)	3.74 (3.85)	0.037	3.78 (4.42)	4.08 (5.36)	3.96 (6.31)	0.431
TG, mmol/L	1.63 (1.27)	1.72 (1.87)	1.79 (1.56)	0.061	1.72 (1.52)	1.74 (1.47)	1.68 (1.75)	0.595	1.69 (1.34)	1.74 (1.96)	1.71 (1.37)	0.736
HDL-C, mmol/L	1.33 (0.38)	1.30 (0.38)	1.27 (0.39)	<0.001	1.34 (0.39)	1.30 (0.38)	1.26 (0.38)	<0.001	1.33 (0.38)	1.30 (0.38)	1.27 (0.39)	<0.001
LDL-C, mmol/L	3.08 (0.95)	3.00 (0.95)	2.96 (0.94)	0.007	2.99 (0.95)	3.01 (0.95)	3.03 (0.94)	0.731	2.99 (0.90)	2.98 (0.96)	3.07 (0.87)	0.035
Total cholesterol, mmol/L	5.17 (1.13)	5.11 (1.16)	5.09 (1.13)	0.089	5.10 (1.14)	5.15 (1.15)	5.11 (1.13)	0.353	5.10 (1.13)	5.12 (1.15)	5.15 (1.15)	0.314
Urinary creatinine, mg/dL	83.7 (62.4)	126.9 (67.2)	151.1 (73.1)	<0.001	69.5 (45.5)	122.2 (58.1)	169.9 (74.5)	<0.001	96.6 (66.4)	124.4 (70.8)	140.6 (75.1)	<0.001
Hypertension, N (%)	1034 (33.9)	1008 (33.0)	1008 (33.0)	0.759	1176 (38.6)	1014 (33.2)	860 (28.2)	<0.001	1116 (36.6)	1035 (33.9)	899 (29.5)	<0.001
Prediabetes, N (%)	1558 (75.6)	1526 (73.4)	1523 (73.6)	<0.001	1417 (68.5)	1545 (74.9)	1645 (79.3)	<0.001	1472 (71.2)	1521 (73.3)	1614 (78.2)	0.212

Continuous variables are presented as mean (SD). Categorical variables are presented as numbers (%, percentage).

Abbreviations: BMI, body mass index; DBP, diastolic blood pressure; FPG, fasting plasma glucose; HDL-C, high-density cholesterol lipoprotein; HOMA-IR, homeostatic model assessment of insulin resistance; LDL-C, low-density cholesterol lipoprotein; SBP, systolic blood pressure; TG, triglycerides.

The differences for the baseline intakes of food groups by tertiles of urinary perchlorate, nitrate, and thiocyanate are presented in Supplementary Table S1 (23). The participants with higher urinary perchlorate had higher intakes of fruits excluding citrus, melons, and berries, cured meat and milk, and the participants with higher urinary nitrate had higher intakes of dark vegetable and other vegetables (excluding tomato, red and orange vegetables, and starchy vegetables) (all *P* < 0.05). The participants with higher urinary thiocyanate had higher intakes of fruits excluding citrus, melons, and berries, total grain, and cured meat (all *P* < 0.05).

### Association of Urinary Perchlorate, Nitrate, and Thiocyanate With Diabetes Complications

Table 2 shows the ORs and 95% CI for the tertiles of urinary perchlorate, nitrate, and thiocyanate in terms of diabetic complications, including congestive heart failure, coronary heart disease, angina, myocardial infarction, stroke, diabetic retinopathy, and diabetic nephropathy. Compared with the lowest tertiles of urinary nitrate, the participants in the highest tertiles had lower risks of congestive heart failure and diabetic nephropathy (OR = 0.41 [95% CI, 0.27-0.60] for congestive heart failure; OR = 0.50 [ 95% CI, 0.41-0.62] for diabetic nephropathy). Meanwhile, no significant association of urinary perchlorate, nitrate, and thiocyanate with other diabetes complications was observed.

**Table 2.** Odds ratios and 95% CI for the association of urinary perchlorate, nitrate, and thiocyanate with the risk of diabetes complications

		Model 1		Model 2		Model 3	
Subgroup	Cases/N	OR (95% CI)	*P*	OR (95% CI)	*P*	OR (95% CI)	*P*
**Congestive heart failure**							
ȃPerchlorate							
ȃȃT1	125/2053	Ref	0.044	Ref	0.020	Ref	0.480
ȃȃT2	104/2067	0.81 (0.61, 1.08)		0.77 (0.57, 1.03)		0.86 (0.64, 1.15)	
ȃȃT3	86/2056	0.67 (0.49, 0.92)		0.63 (0.46, 0.87)		0.83 (0.60, 1.16)	
ȃNitrate							
ȃȃT1	182/2068	Ref	0.001	Ref	0.001	Ref	<0.001
ȃȃT2	74/2045	0.40 (0.29, 0.54)		0.42 (0.31, 0.57)		0.43 (0.32, 0.58)	
ȃȃT3	59/2063	0.33 (0.23, 0.48)		0.38 (0.27, 0.55)		0.42 (0.30, 0.60)	
ȃThiocyanate							
ȃȃT1	134/2053	Ref	0.085	Ref	0.186	Ref	0.654
ȃȃT2	98/2062	0.81 (0.61, 1.07)		0.83 (0.62, 1.10)		0.91 (0.68, 1.22)	
ȃȃT3	83/2061	0.69 (0.49, 0.98)		0.74 (0.52, 1.05)		0.85 (0.59, 1.22)	
**Coronary heart disease**							
ȃPerchlorate							
ȃȃT1	112/2049	Ref	0.262	Ref	0.281	Ref	0.342
ȃȃT2	143/2066	1.18 (0.89, 1.55)		1.14 (0.86, 1.52)		1.15 (0.87, 1.52)	
ȃȃT3	118/2055	0.96 (0.71, 1.30)		0.92 (0.68, 1.25)		0.94 (0.69, 1.28)	
ȃNitrate							
ȃȃT1	154/2062	Ref	0.512	Ref	0.736	Ref	0.370
ȃȃT2	121/2047	0.87 (0.66, 1.15)		0.89 (0.67, 1.18)		0.85 (0.65, 1.11)	
ȃȃT3	98/2061	0.84 (0.61, 1.16)		0.94 (0.68, 1.30)		0.83 (0.61, 1.12)	
ȃThiocyanate							
ȃȃT1	136/2054	Ref	0.595	Ref	0.513	Ref	0.346
ȃȃT2	137/2062	1.14 (0.88, 1.47)		1.17 (0.90, 1.53)		1.22 (0.93, 1.59)	
ȃȃT3	100/2054	1.02 (0.75, 1.41)		1.10 (0.90, 1.52)		1.17 (0.84, 1.63)	
**Angina**							
ȃPerchlorate							
ȃȃT1	95/2072	Ref	0.407	Ref	0.285	Ref	0.414
ȃȃT2	80/2049	0.82 (0.60, 1.13)		0.80 (0.57, 1.10)		0.82 (0.60, 1.13)	
ȃȃT3	78/2060	0.82 (0.58, 1.16)		0.78 (0.55, 1.11)		0.82 (0.58, 1.16)	
ȃNitrate							
ȃȃT1	113/2069	Ref	0.347	Ref	0.597	Ref	0.407
ȃȃT2	78/2051	0.83 (0.60, 1.15)		0.85 (0.62, 1.18)		0.83 (0.60, 1.13)	
ȃȃT3	62/2061	0.77 (0.53, 1.12)		0.86 (0.58, 1.25)		0.83 (0.58, 1.17)	
ȃThiocyanate							
ȃȃT1	103/2059	Ref	0.614	Ref	0.756	Ref	0.912
ȃȃT2	86/2065	0.93 (0.69, 1.26)		0.93 (0.68, 1.27)		0.97 (0.71, 1.33)	
ȃȃT3	64/2057	0.83 (0.57, 1.21)		0.87 (0.59, 1.27)		0.92 (0.62, 1.36)	
**Myocardial infarction**							
ȃPerchlorate							
ȃȃT1	133/2060	Ref	0.858	Ref	0.855	Ref	0.741
ȃȃT2	143/2074	1.06 (0.81, 1.38)		1.03 (0.78, 1.34)		0.97 (0.74, 1.27)	
ȃȃT3	133/2065	0.99 (0.75, 1.32)		0.95 (0.71, 1.27)		0.90 (0.67, 1.20)	
ȃNitrate							
ȃȃT1	177/2076	Ref	0.188	Ref	0.323	Ref	0.044
ȃȃT2	121/2056	0.79 (0.60, 1.02)		0.82 (0.63, 1.07)		0.74 (0.57, 0.95)	
ȃȃT3	111/2067	0.82 (0.61, 1.11)		0.95 (0.70, 1.29)		0.76 (0.57, 1.01)	
ȃThiocyanate							
ȃȃT1	159/2064	Ref	0.317	Ref	0.202	Ref	0.229
ȃȃT2	118/2072	0.86 (0.66, 1.11)		0.83 (0.64, 1.08)		0.84 (0.64, 1.10)	
ȃȃT3	132/2063	1.06 (0.79, 1.42)		1.07 (0.80, 1.45)		1.07 (0.79, 1.46)	
**Stroke**							
ȃPerchlorate							
ȃȃT1	106/2058	Ref	0.342	Ref	0.326	Ref	0.256
ȃȃT2	128/2072	1.18 (0.90, 1.54)		1.19 (0.91, 1.57)		1.27 (0.96, 1.68)	
ȃȃT3	107/2065	0.98 (0.74, 1.31)		1.00 (0.75, 1.33)		1.13 (0.83, 1.56)	
ȃNitrate							
ȃȃT1	152/2065	Ref	0.065	Ref	0.357	Ref	0.509
ȃȃT2	105/2062	0.82 (0.63, 1.07)		0.86 (0.66, 1.13)		0.88 (0.67, 1.16)	
ȃȃT3	84/2068	0.72 (0.54, 0.95)		0.82 (0.61, 1.10)		0.85 (0.62, 1.15)	
ȃThiocyanate							
ȃȃT1	147/2064	Ref	0.068	Ref	0.092	Ref	0.217
ȃȃT2	105/2072	0.80 (0.61, 1.04)		0.79 (0.61, 1.04)		0.83 (0.63, 1.10)	
ȃȃT3	89/2059	0.70 (0.50, 0.97)		0.72 (0.52, 1.00)		0.76 (0.54, 1.07)	
**Diabetic Retinopathy**							
ȃPerchlorate							
ȃȃT1	135/527	Ref	0.058	Ref	0.048	Ref	0.149
ȃȃT2	120/526	0.94 (0.70, 1.26)		0.94 (0.70, 1.27)		0.95 (0.71, 1.28)	
ȃȃT3	92/529	0.69 (0.50, 0.95)		0.68 (0.49, 0.95)		0.73 (0.53, 1.02)	
ȃNitrate							
ȃȃT1	145/525	Ref	0.005	Ref	0.012	Ref	0.029
ȃȃT2	109/529	0.69 (0.51, 0.93)		0.69 (0.51, 0.94)		0.74 (0.55, 0.97)	
ȃȃT3	93/528	0.58 (0.41, 0.82)		0.61 (0.43, 0.86)		0.66 (0.47, 0.93)	
ȃThiocyanate							
ȃȃT1	141/526	Ref	0.006	Ref	0.012	Ref	0.100
ȃȃT2	109/530	0.72 (0.54, 0.97)		0.74 (0.55, 1.00)		0.85 (0.64, 1.14)	
ȃȃT3	97/526	0.60 (0.43, 0.83)		0.61 (0.43, 0.86)		0.67 (0.46, 0.97)	
**Diabetic nephropathy**							
ȃPerchlorate							
ȃȃT1	557/2061	Ref	0.148	Ref	0.053	Ref	0.492
ȃȃT2	555/2078	0.97 (0.83, 1.13)		0.95 (0.81, 1.12)		1.09 (0.93, 1.28)	
ȃȃT3	504/2069	0.86 (0.73, 1.01)		0.82 (0.69, 0.97)		1.93 (0.92, 1.30)	
ȃNitrate							
ȃȃT1	742/2070	Ref	0.001	Ref	0.001	Ref	0.001
ȃȃT2	499/2064	0.63 (0.54, 0.73)		0.66 (0.56, 0.78)		0.73 (0.63, 0.86)	
ȃȃT3	375/2057	0.45 (0.38, 0.54)		0.49 (0.40, 0.59)		0.59 (0.50, 0.70)	
ȃThiocyanate							
ȃȃT1	674/2068	Ref	0.001	Ref	0.001	Ref	0.065
ȃȃT2	540/2075	0.87 (0.75, 1.01)		0.87 (0.74, 1.01)		0.95 (0.81, 1.10)	
ȃȃT3	402/2065	0.69 (0.58, 0.83)		0.70 (0.58, 0.84)		0.80 (0.66, 0.97)	

Data are ORs and 95%CI.

Model 1 was adjusted for age, gender, race, education, income, smoking, drinking, regular exercise habits, body mass index, daily energy intake, and Alternative Healthy Eating Index.

Model 2 was model 1 with additional adjustment for drug use for controlling hyperglycemia, hypertension and dyslipidemia, SBP, DBP, urinary creatinine, fasting plasma glucose, HbA1c, HOMA-IR, TG, HDL-C, LDL-C, and TC.

Model 3 was model 2 with mutual adjustment for urinary perchlorate, nitrate, and thiocyanate.

There are 32 participants, 38 participants, 27 participants, 9 participants, 13 participants, and 4626 participants, respectively, who lacked the information of congestive heart failure, coronary heart disease, angina, stroke, myocardial infarction, and diabetic retinopathy. Therefore, we excluded these participants when analyzing the specific diabetes complications.

### Association of Urinary Perchlorate, Nitrate, and Thiocyanate With All-Cause and Disease-Specific Mortality

The hazard ratios (HRs) and 95% CI for the association of tertiles of urinary perchlorate, nitrate, thiocyanate with mortalities of all-cause, CVD, diabetes, and cancer were presented in Table 3. Compared with the lowest tertiles of urinary nitrate, the participants in the highest tertiles had lower mortalities of all-cause (HR = 0.78; 95% CI, 0.62-0.97), CVD (HR = 0.56; 95% CI, 0.37-0.84) and diabetes (HR = 0.47; 95% CI, 0.24-0.90), and no significant association between urinary nitrate and cancer mortality was observed (HR = 1.13; 95% CI, 0.71-1.80). Also, no significant association of urinary perchlorate and thiocyanate with total and disease-specific mortality was observed.

**Table 3.** HRs and 95%CI for the association of urinary perchlorate, nitrate, and thiocyanate with all-cause and disease-specific mortalities

		Model 1		Model 2		Model 3	
Subgroup	Cases/N	HR (95% CI)	*P*	HR (95% CI)	*P*	HR (95% CI)	*P*
**All-cause mortality**							
ȃPerchlorate							
ȃȃT1	270/2061	Ref	0.685	Ref	0.531	Ref	0.835
ȃȃT2	277/2078	0.94 (0.79,1.12)		0.92 (0.77,1.10)		0.95 (0.80,1.13)	
ȃȃT3	269/2069	0.93 (0.77,1.12)		0.90 (0.75,1.09)		0.98 (0.81,1.18)	
ȃNitrate							
ȃȃT1	373/2070	Ref	0.009	Ref	0.035	Ref	0.017
ȃȃT2	245/2064	0.82 (0.69,0.97)		0.86 (0.72,1.02)		0.85 (0.72,1.01)	
ȃȃT3	198/2074	0.74 (0.60,0.91)		0.77 (0.63,0.95)		0.76 (0.63,0.92)	
ȃThiocyanate							
ȃȃT1	332/2068	Ref	0.991	Ref	0.335	Ref	0.568
ȃȃT2	248/2075	0.89 (0.76,1.06)		0.88 (0.75,1.04)		0.91 (0.77,1.08)	
ȃȃT3	236/2065	0.92 (0.75,1.12)		0.92 (0.76,1.13)		0.96 (0.79,1.18)	
**CVD mortality**							
ȃPerchlorate							
ȃȃT1	84/2061	Ref	0.228	Ref	0.240	Ref	0.319
ȃȃT2	94/2078	1.04 (0.77,1.42)		1.02 (0.75,1.40)		1.03 (0.76,1.40)	
ȃȃT3	70/2069	0.80 (0.56,1.13)		0.79 (0.55,1.12)		0.81 (0.57,1.15)	
ȃNitrate							
ȃȃT1	130/2070	Ref	0.004	Ref	0.011	Ref	0.016
ȃȃT2	68/2064	0.66 (0.48,0.91)		0.70 (0.51,0.96)		0.72 (0.53,0.98)	
ȃȃT3	50/2074	0.55 (0.38,0.81)		0.58 (0.39,0.85)		0.61 (0.42,0.88)	
ȃThiocyanate							
ȃȃT1	100/2068	Ref	0.700	Ref	0.494	Ref	0.476
ȃȃT2	75/2075	0.92 (0.68,1.24)		0.90 (0.66,1.22)		0.93 (0.68,1.26)	
ȃȃT3	73/2065	1.12 (0.78,1.59)		1.11 (0.78,1.59)		1.17 (0.81,1.69)	
**Diabetes mortality**							
ȃPerchlorate							
ȃȃT1	46/2061	Ref	0.954	Ref	0.913	Ref	0.521
ȃȃT2	39/2078	0.94 (0.60,1.47)		0.83 (0.52,1.30)		0.86 (0.55,1.35)	
ȃȃT3	38/2069	0.99 (0.61,1.60)		0.91 (0.56,1.47)		1.13 (0.69,1.84)	
ȃNitrate							
ȃȃT1	66/2070	Ref	0.035	Ref	0.023	Ref	0.008
ȃȃT2	40/2064	0.87 (0.57,1.34)		0.95 (0.62,1.46)		0.90 (0.60,1.36)	
ȃȃT3	17/2057	0.45 (0.24,0.83)		0.44 (0.23,0.81)		0.39 (0.22,0.71)	
ȃThiocyanate							
ȃȃT1	50/2068	Ref	0.018	Ref	0.011	Ref	0.017
ȃȃT2	50/2075	1.34 (0.90,2.00)		1.29 (0.86,1.93)		1.35 (0.90,2.02)	
ȃȃT3	23/2065	0.60 (0.33,1.07)		0.54 (0.29,0.97)		0.58 (0.31,1.07)	
**Cancer mortality**							
ȃPerchlorate							
ȃȃT1	68/2061	Ref	0.157	Ref	0.144	Ref	0.195
ȃȃT2	57/2078	0.70 (0.48,1.01)		0.70 (0.48,1.01)		0.71 (0.50,1.03)	
ȃȃT3	66/2069	0.79 (0.54,1.15)		0.76 (0.52,1.11)		0.83 (0.57,1.21)	
ȃNitrate							
ȃȃT1	69/2070	Ref	0.654	Ref	0.005	Ref	0.484
ȃȃT2	68/2064	1.11 (0.78,1.58)		1.13 (0.79,1.62)		1.26 (0.86,1.83)	
ȃȃT3	54/2057	0.94 (0.62,1.43)		0.98 (0.64,1.49)		1.13 (0.71,1.80)	
ȃThiocyanate							
ȃȃT1	70/2068	Ref	0.783	Ref	0.818	Ref	0.812
ȃȃT2	57/2075	0.89 (0.62,1.27)		0.89 (0.63,1.28)		0.89 (0.63,1.28)	
ȃȃT3	64/2065	0.90 (0.60,1.35)		0.93 (0.62,1.39)		0.91 (0.60,1.38)	

Data are hazard ratios and 95% CI.

Model 1 was adjusted for age, gender, race, education, income, smoking, drinking, regular exercise habits, body mass index, daily energy intake, and Alternative Healthy Eating Index.

Model 2 was model 1 with additional adjustment for drug use for controlling hyperglycemia, hypertension and dyslipidemia, SBP, DBP, urinary creatinine, fasting plasma glucose, HbA1c, HOMA-IR, TG, HDL-C, LDL-C, and TC.

Model 3 was model 2 with mutual adjustment for urinary perchlorate, nitrate, thiocyanate, and serum cotinine level.

### Restricted Cubic Spline for the HRs and 95% CI in Terms of Association of Urinary Nitrate With Diabetes Complication and Mortality

**Figure 1.**
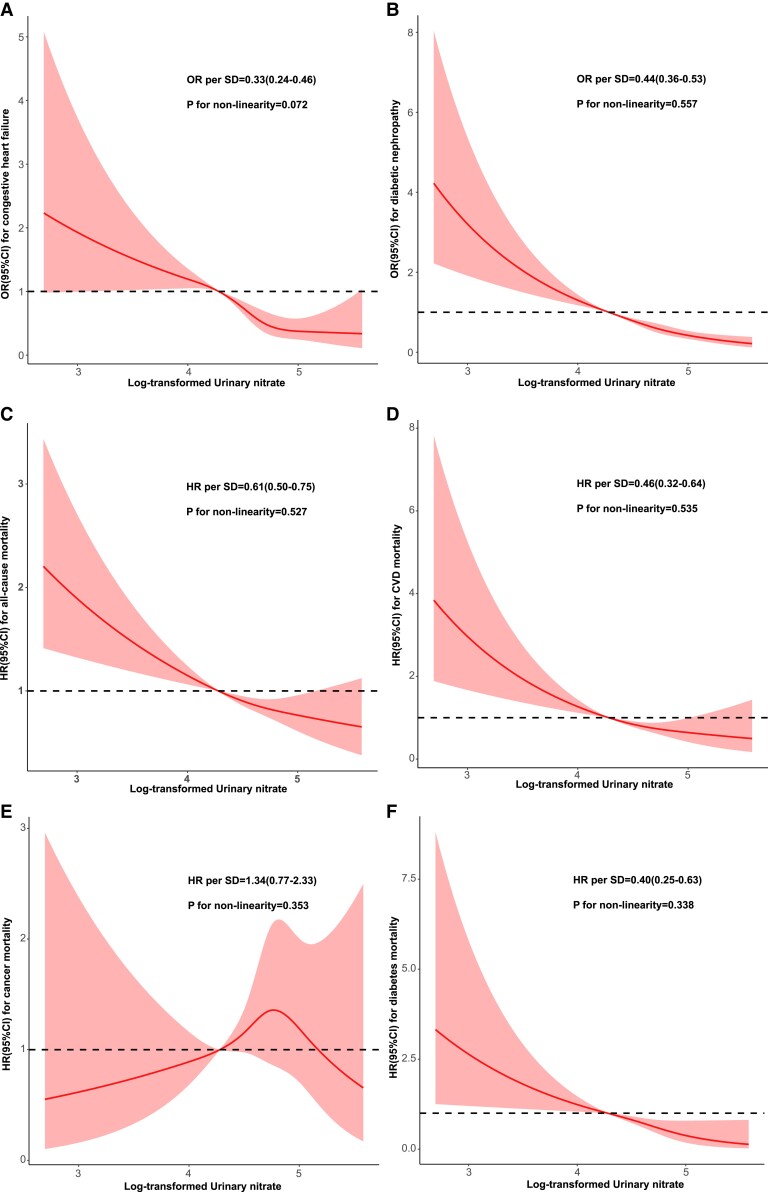
Restricted cubic spline analysis between log-transformed urinary nitrate and the risk of congestive heart failure (a), diabetic nephropathy (b), all-cause mortality (c), CVD mortality (d), cancer mortality (e), and diabetes mortality (f). Abbreviations: HR, hazard ratio; OR, odds ratio.

Due to the significant negative association of tertiles of urinary nitrate with the risk of congestive heart failure and diabetic nephropathy, and also mortality from all causes, CVD, and diabetes documented above, the restricted cubic spline was used to flexibly model for visualizing the above association, which is presented in Fig. 1. Urinary nitrate showed linear relationships with congestive heart failure and diabetic nephropathy, and deaths from all causes, CVD, and diabetes (all *P* for nonlinearity >0.05). The ORs per SD higher predicted risk of congestive heart failure and diabetic nephropathy were 0.33 (0.24-0.46) and 0.44 (0.36-0.53), respectively. The HRs per SD higher predicted risks of all-cause, CVD and diabetes mortality were 0.61 (0.50-0.75), 0.46 (0.32-0.64), and 0.40 (0.25-0.63).

### Sensitivity Analysis

In the first sensitivity analysis, after exclusion the participants who had a follow-up time of less than 2 years, the association of urinary nitrate with congestive heart failure, diabetic nephropathy, and CVD mortality were still observed, suggesting that the serious illness might not influence the above association (Supplementary Tables S2 and S3) (23). The second sensitivity analysis indicated that extreme values of urinary perchlorate, nitrate, and thiocyanate did not influence the above results (Supplementary Tables S4 and S5) (23). The third sensitivity analysis showed that after corrected by urinary creatinine, the results are also robust (Supplementary Tables S6 and S7) (23). The fourth sensitivity analysis showed BMI, smoking, and drinking did not modify the relationships of urinary nitrate with congestive heart failure, diabetic nephropathy, and all-cause, diabetes, and CVD mortality (Supplementary Tables S8 and S9) (23). Moreover, we observed that smoking status had a modification effect on the association of urinary nitrate with coronary heart disease and myocardial infarction (Supplementary Table S10) (23). Results showed a significant opposite association between urinary nitrate and coronary heart disease and myocardial infarction in smoking participants to that in nonsmoking participants, likely because smoking could significantly increase the circulating levels of thiocyanate, which might influence nitrate metabolism by their internal competitive effects. Besides, we also found that the partial interaction impacts of age and gender on the association of urinary nitrate with disease-specific and all-cause mortality had marginal significance. Additionally, the results of stratification analyses by age and gender indicated that inorganic nitrate supplementation to the male and elder population with hyperglycemia should be prioritized (Supplementary Tables S11-S18) (23).

## Discussion

This study examined the association of urinary perchlorate, nitrate, and thiocyanate with the risk of diabetes complications, disease-specific mortality, and all-cause mortality among participants with hyperglycemia. This study found that participants with higher urinary nitrate levels had a lower risk of congestive heart failure and diabetic nephropathy, as well as lower mortality from all causes, CVD, and diabetes among the participants with hyperglycemia. Moreover, no significant association between urinary nitrate and cancer mortality was observed, and the participants with higher urinary nitrate consumed more dark vegetables and other vegetables (excluding tomato, red and orange vegetables, and starchy vegetables).

To date, it is still largely unknown whether urinary nitrate levels are associated with diabetes complications and long-term survival among patients with hyperglycemia although elucidating this association may add important knowledge regarding the health impacts of nitrate on the natural course of diabetes. Therefore, the most important finding in this study was that the participants with higher urinary levels had a lower risk of congestive heart failure and diabetic nephropathy, and lower risk of all-cause, CVD, and diabetes mortality, and the above association consistently showed a linear dose-dependent manner. These findings could be partially supported by previous studies. It has been documented that urinary nitrate is a reliable marker to reflect the dietary nitrate intake (3), and a few observational studies among the general population have already found that higher dietary nitrate intake was associated with a lower incidence of CVD and CVD mortality (24, 25). For the evidence of urinary nitrate, a recent cross-sectional study among the general population showed that higher urinary nitrate levels were associated with a lower risk of congestive heart failure (26), and patients with diabetes had lower levels of urinary nitrate, especially in diabetic nephropathy (27-29). Meanwhile, the association between higher urinary nitrate and lower CVD mortality among the general population has also been reported (30), and a few randomized controlled clinical trials have also shown that nitrate supplementation could lower blood pressure, improve vascular function, enhance exercise capacity, and reduce renal resistive index among patients with hypertension, heart failure, or hypertension- related nephropathies (31-34).

Elucidating the mechanism, a few vivo and vitro studies have examined the health effects of nitrate in the models of hyperglycemic condition, finding that nitrate supplementation could increase pancreatic islet blood flow and insulin secretion through modulation of reactive oxygen species (ROS) signaling with an improvement of glucose tolerance and lipid profile (35), and it could normalize the recovery of the hearts after ischemia-reperfusion injury and restore ischemic hind limb blood flow in a VEGF-dependent, NO-mediated manner (36). Also, it has been found that administration of nitrate in diet could improve kidney function and have beneficial renovascular effects in aged rats with metabolic syndrome and reduced kidney function by reduction of oxidative stress and restoration of NO homeostasis in the kidney (37, 38). And for both metabolic disorders and chronic hyperglycemia, it has been shown that the imbalance between oxidative stress and impaired of the NOX activity with subsequent NO deficiency are closely related to the progression of both macro- and microvascular dysfunction. Accumulating evidence has suggested that stimulating the nitrate-nitrite-NO pathway, which could be enhanced by dietary nitrate supplementation, contributes to restoring REDOX state imbalance and NO bioavailability by reducing mitochondria-derived ROS (the interaction between the nitrate-derived reactive nitrogen oxide species, mitochondria, and NADPH enzymes), thereby improving vasodilation, inhibiting small resistance artery constriction, and decreasing plasma creatinine. Moreover, previous studies have documented that the NO homeostasis of the normal population is different from the population with hyperglycemia. Compared with a healthy condition, long-term hyperglycemic exposure can increase the excessive accumulation of superoxide by inducing mitochondrial ROS production, which is the main cause of NO homeostasis disorder, through 3 major mechanisms. The first is that the accumulation of peroxides inhibits endothelial nitric oxide synthase activity through posttranslational modification of the Akt site, contributing to decreasing the production of NO (39). Secondly, the content of NO is also decreased due to its oxidation by the superoxide anion (O_2_^−^) to peroxynitrite (ONOO^−^) (40). Lastly, the nitrosylation of proteins caused by the peroxynitrite in turn inhibits the activities of antioxidant enzymes and endothelial nitric oxide synthase, thus forming a vicious cycle among the condition of hyperglycemia (41). These studies provided the potential mechanisms for the association of higher urinary nitrate with lower risks of congestive heart failure and diabetic nephropathy, and the lower mortality from all causes, CVD, and diabetes documented in this study. Taken together, the findings in this study and the current evidence from human and animal studies collectively indicated the potential beneficial health effects on the complications of diabetes and long-term survival among patients with hyperglycemia.

Moreover, this study did not observe the association between urinary nitrate and cancer mortality. Although it has been demonstrated that nitrate intake can form nitrosating agents in the acidic stomach and therefore produce potentially carcinogenic nitrosamines (42), which emphasizes the importance of limiting the levels of nitrate in drinking water and food, the current evidence from human studies regarding this issue is inconsistent (43). Further, the recent observational study among the general population reported an association between higher urinary nitrate and greater all-cause and cancer mortality (44), which was inconsistent with the findings in this study. The different sources of dietary nitrate may be a possible explanation for these inconsistent results. The previous study found that dietary nitrate from processed meat was associated with the incidence of cancer, whereas the dietary nitrate from vegetables was not (45). Consistent with this result, this study also found that participants with higher urinary nitrate levels had a higher intake of dark vegetables and other vegetables (excluding tomato, red and orange vegetables, and starchy vegetables). Meanwhile, the International Agency for Research on Cancer (IARC) and the European Food Safety Authority (EFSA) also stated that there is inadequate evidence in humans for the carcinogenicity of nitrate in food and drinking water (46, 47).

### Strengths and Limitations

This study had several strengths. This study is the first to examine the association of urinary nitrate with the risk of diabetes complications and all-cause, CVD, diabetes, and cancer mortality, with simultaneously considering the urinary levels of perchlorate and thiocyanate because of the competitive effects. Moreover, the association reported in this study was relatively robust, with adjustment for a series of classic confounders that are associated with the diabetes complication and long-term survival among patients with hyperglycemia. However, this study has certain limitations. First, the data evaluating the association between urinary nitrate and diabetes complications were cross-sectional. Although the association between nitrate and mortality may affect this limitation, future study is needed to examine the association between nitrate and incidence of diabetes complications. Second, this study lacked the information for distinguishing the cause of hyperglycemia by the type of diabetes. Although the previous study reported that most of the participants with diabetes in the NHANES population had type 2 diabetes (48), future study is needed to examine this association with regard to different types of diabetes to provide more comprehensive evidence. Third, this study is observational in nature. Although we have already adjusted most known confounders, we cannot exclude the possibility that unmeasured variables may influence our results. Fourth, the urinary perchlorate, nitrate, and thiocyanate were only measured at baseline, thus may not provide information regarding health impacts of dynamic changes in these biomarkers; future study is warranted to evaluate the longitudinal effect of urinary nitrate on long-term survival among patients with hyperglycemia.

### Clinical Implications

The findings of this study have important clinical implications. This study provided relatively comprehensive evidence for the beneficial effects of circulating urinary nitrate among patients with hyperglycemia. Also, this study found that higher urinary nitrate was not associated with cancer mortality, although perhaps due to the relatively short duration of follow-up. This information is of importance in providing effective treatment plans for patients with hyperglycemia using natural sources of nitrate for decreasing the risk of diabetes complications and improving long-term survival. Besides, the results of the stratification analysis also indicated that the public health system may consider focusing on inorganic nitrate supplementation to the male and elder population with hyperglycemia.

## Conclusion

In conclusion, higher urinary nitrate is associated with a lower risk of congestive heart failure and diabetic nephropathy, as well as lower mortality from all causes, CVD, and diabetes. No significant association between urinary nitrate with cancer mortality was observed. These findings indicate that inorganic nitrate supplementation can be considered as a supplementary treatment for patients with hyperglycemia, prioritizing the male and elder population with hyperglycemia.

## Data Availability

All the supplementary data generated or analyzed in this study are included in the cited dataset (23) (Jiang, Wenbo, 2022, “Supplementary tables of the association of urinary nitrate with the risk of diabetes complications and disease-specific mortalities among adults with hyperglycemia. DRAFT VERSION ed: Harvard dataverse; 2022.”, https://doi.org/10.7910/DVN/MPPJTC).

## Abbreviations

AHEI: Alternative Healthy Eating Index
BMI: body mass index
CVD: cardiovascular disease
DBP: diastolic blood pressure
HbA1c: glycosylated hemoglobin, type A1
HDL-C: high-density lipoprotein cholesterol
HR: hazard ratio
HOMA-IR: homeostatic model assessment of insulin resistance
LDL-C: low-density lipoprotein cholesterol
LOD: limits of detection
NHANES: National Health and Nutrition Examination Survey
NO: nitric oxide
OR: odds ratio
ROS: reactive oxygen species
SBP: systolic blood pressure
TC: total cholesterol
TG: triglycerides

## References

[1] Sindelar JJ , Milkowski AL. Human safety controversies surrounding nitrate and nitrite in the diet. Nitric Oxide. 2012;26(4):259‐266. 22487433 10.1016/j.niox.2012.03.011

[2] Hord NG , Tang Y, Bryan NS. Food sources of nitrates and nitrites: the physiologic context for potential health benefits. Am J Clin Nutr. 2009;90(1):1‐10. 19439460 10.3945/ajcn.2008.27131

[3] Bedale W , Sindelar JJ, Milkowski AL. Dietary nitrate and nitrite: benefits, risks, and evolving perceptions. Meat Sci. 2016;120:85‐92. 26994928 10.1016/j.meatsci.2016.03.009

[4] Tsikas D . Analysis of nitrite and nitrate in biological fluids by assays based on the Griess reaction: appraisal of the Griess reaction in the L-arginine/nitric oxide area of research. J Chromatogr B Analyt Technol Biomed Life Sci. 2007;851(1-2):51‐70. 10.1016/j.jchromb.2006.07.054 16950667

[5] Ellis G , Adatia I, Yazdanpanah M, Makela SK. Nitrite and nitrate analyses: a clinical biochemistry perspective. Clin Biochem. 1998;31(4):195‐220. 9646943 10.1016/s0009-9120(98)00015-0

[6] Suh M , Abraham L, Hixon JG, Proctor DM. The effects of perchlorate, nitrate, and thiocyanate on free thyroxine for potentially sensitive subpopulations of the 2001-2002 and 2007-2008 National Health and Nutrition Examination Surveys. J Expo Sci Environ Epidemiol. 2014;24(6):579‐587. 24149973 10.1038/jes.2013.67

[7] Wu Z , Jin T, Weng J. A thorough analysis of diabetes research in China from 1995 to 2015: current scenario and future scope. Sci China Life Sci. 2019;62(1):46‐62. 30267261 10.1007/s11427-018-9377-y

[8] Bahadoran Z , Ghasemi A, Mirmiran P, Azizi F, Hadaegh F. Beneficial effects of inorganic nitrate/nitrite in type 2 diabetes and its complications. Nutr Metab (Lond). 2015;12(1):16. 25991919 10.1186/s12986-015-0013-6 PMC4436104

[9] Beckman JA , Paneni F, Cosentino F, Creager MA. Diabetes and vascular disease: pathophysiology, clinical consequences, and medical therapy: part II. Eur Heart J. 2013;34(31):2444‐2452. 23625211 10.1093/eurheartj/eht142

[10] Eid AA , Lee DY, Roman LJ, Khazim K, Gorin Y. Sestrin 2 and AMPK connect hyperglycemia to Nox4-dependent endothelial nitric oxide synthase uncoupling and matrix protein expression. Mol Cell Biol. 2013;33(17):3439‐3460. 23816887 10.1128/MCB.00217-13 PMC3753845

[11] Bryan NS , Fernandez BO, Bauer SM, et al Nitrite is a signaling molecule and regulator of gene expression in mammalian tissues. Nat Chem Biol. 2005;1(5):290‐297. 16408059 10.1038/nchembio734

[12] Bahadoran Z , Norouzirad R, Mirmiran P, et al Effect of inorganic nitrate on metabolic parameters in patients with type 2 diabetes: a 24-week randomized double-blind placebo-controlled clinical trial. Nitric Oxide. 2021;107:58‐65. 33340674 10.1016/j.niox.2020.12.005

[13] Floyd CN , Lidder S, Hunt J, Omar SA, McNeill K, Webb AJ. Acute interaction between oral glucose (75 g as Lucozade) and inorganic nitrate: decreased insulin clearance, but lack of blood pressure-lowering. Br J Clin Pharmacol. 2019;85(7):1443‐1453. 30845346 10.1111/bcp.13913 PMC6595348

[14] Morselli F , Faconti L, Mills CE, et al Dietary nitrate prevents progression of carotid subclinical atherosclerosis through blood pressure-independent mechanisms in patients with or at risk of type 2 diabetes mellitus. Br J Clin Pharmacol. 2021;87(12):4726‐4736. 33982797 10.1111/bcp.14897

[15] Henstridge DC , Kingwell BA, Formosa MF, Drew BG, McConell GK, Duffy SJ. Effects of the nitric oxide donor, sodium nitroprusside, on resting leg glucose uptake in patients with type 2 diabetes. Diabetologia. 2005;48(12):2602‐2608. 16273348 10.1007/s00125-005-0018-1

[16] Gilchrist M , Winyard PG, Aizawa K, Anning C, Shore A, Benjamin N. Effect of dietary nitrate on blood pressure, endothelial function, and insulin sensitivity in type 2 diabetes. Free Radic Biol Med. 2013;60:89‐97. 23395779 10.1016/j.freeradbiomed.2013.01.024

[17] Gilchrist M , Winyard PG, Fulford J, Anning C, Shore AC, Benjamin N. Dietary nitrate supplementation improves reaction time in type 2 diabetes: development and application of a novel nitrate-depleted beetroot juice placebo. Nitric Oxide. 2014;40:67‐74. 24858657 10.1016/j.niox.2014.05.003

[18] Shan Z , Rehm CD, Rogers G, et al Trends in dietary carbohydrate, protein, and fat intake and diet quality among US adults, 1999-2016. JAMA. 2019;322(12):1178‐1187. 31550032 10.1001/jama.2019.13771 PMC6763999

[19] American Diabetes Association Professional Practice Committee . 2. Classification and diagnosis of diabetes: standards of medical care in diabetes-2022. Diabetes Care. 2022;45(Suppl_1):S17‐S38. 34964875 10.2337/dc22-S002

[20] Blount BC , Valentin-Blasini L, Osterloh JD, Mauldin JP, Pirkle JL. Perchlorate exposure of the US population, 2001-2002. J Expo Sci Environ Epidemiol. 2007;17(4):400‐407. 17051137 10.1038/sj.jes.7500535

[21] Levey AS , Stevens LA, Schmid CH, et al A new equation to estimate glomerular filtration rate. Ann Intern Med. 2009;150(9):604‐612. 19414839 10.7326/0003-4819-150-9-200905050-00006 PMC2763564

[22] de Boer IH , Rue TC, Hall YN, Heagerty PJ, Weiss NS, Himmelfarb J. Temporal trends in the prevalence of diabetic kidney disease in the United States. JAMA. 2011;305(24):2532‐2539. 21693741 10.1001/jama.2011.861 PMC3731378

[23] Jiang W . Supplementary tables of the association of urinary nitrate with the risk of diabetes complications and disease-specific mortalities among adults with hyperglycemia. DRAFT VERSION ed: Harvard dataverse; 2022; Deposited November 30, 2022. 10.7910/DVN/MPPJTC

[24] Liu AH , Bondonno CP, Russell J, et al Relationship of dietary nitrate intake from vegetables with cardiovascular disease mortality: a prospective study in a cohort of older Australians. Eur J Nutr. 2019;58(7):2741‐2753. 30238316 10.1007/s00394-018-1823-x

[25] Bondonno CP , Dalgaard F, Blekkenhorst LC, et al Vegetable nitrate intake, blood pressure and incident cardiovascular disease: Danish diet, cancer, and health study. Eur J Epidemiol. 2021;36(8):813‐825. 33884541 10.1007/s10654-021-00747-3 PMC8416839

[26] Wu Z , Tian T, Ma W, Gao W, Song N. Higher urinary nitrate was associated with lower prevalence of congestive heart failure: results from NHANES. BMC Cardiovasc Disord. 2020;20(1):498. 33238887 10.1186/s12872-020-01790-w PMC7690024

[27] Sokolovska J , Dekante A, Baumane L, et al Nitric oxide metabolism is impaired by type 1 diabetes and diabetic nephropathy. Biomed Rep. 2020;12(5):251‐258. 32257188 10.3892/br.2020.1288 PMC7100134

[28] Tessari P , Cecchet D, Cosma A, et al Nitric oxide synthesis is reduced in subjects with type 2 diabetes and nephropathy. Diabetes. 2010;59(9):2152‐2159. 20484137 10.2337/db09-1772 PMC2927936

[29] Imanishi M , Okada N, Konishi Y, et al Angiotensin II receptor blockade reduces salt sensitivity of blood pressure through restoration of renal nitric oxide synthesis in patients with diabetic nephropathy. J Renin Angiotensin Aldosterone Syst. 2013;14(1):67‐73. 22859713 10.1177/1470320312454764

[30] Mendy A . Association of urinary nitrate with lower prevalence of hypertension and stroke and with reduced risk of cardiovascular mortality. Circulation. 2018;137(21):2295‐2297. 29784682 10.1161/CIRCULATIONAHA.118.034168

[31] Falls R , Seman M, Braat S, Sortino J, Allen JD, Neil CJ. Inorganic nitrate as a treatment for acute heart failure: a protocol for a single center, randomized, double-blind, placebo-controlled pilot and feasibility study. J Transl Med. 2017;15(1):172. 28789663 10.1186/s12967-017-1271-z PMC5549289

[32] Kapil V , Khambata RS, Robertson A, Caulfield MJ, Ahluwalia A. Dietary nitrate provides sustained blood pressure lowering in hypertensive patients: a randomized, phase 2, double-blind, placebo-controlled study. Hypertension. 2015;65(2):320‐327. 25421976 10.1161/HYPERTENSIONAHA.114.04675 PMC4288952

[33] Kemmner S , Lorenz G, Wobst J, et al Dietary nitrate load lowers blood pressure and renal resistive index in patients with chronic kidney disease: a pilot study. Nitric Oxide. 2017;64:7‐15. 28137609 10.1016/j.niox.2017.01.011

[34] Nyberg M , Christensen PM, Blackwell JR, Hostrup M, Jones AM, Bangsbo J. Nitrate-rich beetroot juice ingestion reduces skeletal muscle O(2) uptake and blood flow during exercise in sedentary men. J Physiol. 2021;599(23):5203‐5214. 34587650 10.1113/JP281995

[35] Nyström T , Ortsäter H, Huang Z, et al Inorganic nitrite stimulates pancreatic islet blood flow and insulin secretion. Free Radic Biol Med. 2012;53(5):1017‐1023. 22750508 10.1016/j.freeradbiomed.2012.06.031

[36] Bir SC , Pattillo CB, Pardue S, et al Nitrite anion therapy protects against chronic ischemic tissue injury in db/db diabetic mice in a NO/VEGF-dependent manner. Diabetes. 2014;63(1):270‐281. 24009258 10.2337/db13-0890 PMC4179307

[37] Carvalho L , Guimarães DD, Flôr AFL, et al Effects of chronic dietary nitrate supplementation on longevity, vascular function and cancer incidence in rats. Redox Biol. 2021;48:102209. 10.1016/j.redox.2021.102209 PMC8683768 34915448

[38] Carlström M . Nitric oxide signalling in kidney regulation and cardiometabolic health. Nat Rev Nephrol. 2021;17(9):575‐590. 34075241 10.1038/s41581-021-00429-z PMC8169406

[39] Dossumbekova A , Berdyshev EV, Gorshkova I, et al Akt activates NOS3 and separately restores barrier integrity in H2O2-stressed human cardiac microvascular endothelium. Am J Physiol Heart Circ Physiol. 2008;295(6):H2417‐H2426. 18931031 10.1152/ajpheart.00501.2008 PMC2614535

[40] Korda M , Kubant R, Patton S, Malinski T. Leptin-induced endothelial dysfunction in obesity. Am J Physiol Heart Circ Physiol. 2008;295(4):H1514‐H1521. 18689498 10.1152/ajpheart.00479.2008 PMC2593507

[41] Miles JA , Egan JL, Fowler JA, et al The evolutionary origins of peroxynitrite signalling. Biochem Biophys Res Commun. 2021;580:107‐112. 34638028 10.1016/j.bbrc.2021.09.071

[42] Bruning-Fann CS , Kaneene JB. The effects of nitrate, nitrite and N-nitroso compounds on human health: a review. Vet Hum Toxicol. 1993;35(6):521‐538. 8303822

[43] Abasse KS , Essien EE, Abbas M, et al Association between dietary nitrate, nitrite intake, and site-specific cancer risk: a systematic review and meta-analysis. Nutrients. 2022;14(3):666. 35277025 10.3390/nu14030666 PMC8838348

[44] Wang L , Fu Z, Gao B, Mo X, Liang P, Huang J. The association between environmental exposure to perchlorate, nitrate, and thiocyanate and all-cause and cause-specific mortality. Environ Sci Pollut Res Int. 2022;29(15):21851‐21859. 34773236 10.1007/s11356-021-17423-4

[45] Kotopoulou S , Zampelas A, Magriplis E. Dietary nitrate and nitrite and human health: a narrative review by intake source. Nutr Rev. 2022;80(4):762‐773. 34919725 10.1093/nutrit/nuab113

[46] IARC monographs on the evaluation of carcinogenic risks to humans . Ingested nitrate and nitrite, and cyanobacterial peptide toxins. IARC Monogr Eval Carcinog Risks Hum. 2010;94:v‐vii. 1-412. 21141240 PMC4781178

[47] European Food Safety Authority . EFSA explains risk assessment: nitrites and nitrates added to food. Accessed June 15, 2017. https://www.efsa.europa.eu/en/corporate/pub/nitritesandnitrates170614

[48] Berkowitz SA , Meigs JB, Wexler DJ. Age at type 2 diabetes onset and glycaemic control: results from the National Health and Nutrition Examination Survey (NHANES) 2005-2010. Diabetologia. 2013;56(12):2593‐2600. 23995472 10.1007/s00125-013-3036-4 PMC3818392

